# Temporal Patterns and Comparative Analysis of Emergency Department Utilization for Abdominal Paracentesis: Insights From the National Emergency Department Sample Data (2012-2021)

**DOI:** 10.7759/cureus.97503

**Published:** 2025-11-22

**Authors:** Nofel Iftikhar, Ali Aalam

**Affiliations:** 1 Biology, University of Florida, Gainesville, USA; 2 Epidemiology, University of Florida, Gainesville, USA

**Keywords:** abdominal paracentesis, acute alcoholic hepatitis, adult gastroenterology, emergency room (er), hepatology

## Abstract

Introduction

Abdominal paracentesis is a commonly performed diagnostic and therapeutic procedure for the management of ascites, most often associated with liver cirrhosis and alcoholic hepatitis (LCAH). Understanding how its use in the emergency department (ED) has changed over time can help optimize resource allocation and patient care. This study used National Emergency Department Sample (NEDS) data to describe temporal trends in ED presentations for LCAH and associated abdominal paracenteses in the United States from 2012 to 2021.

Methods

We conducted a retrospective observational study of adult ED encounters in NEDS between 2012 and 2021. Encounters with a primary or secondary diagnosis of LCAH were identified using International Classification of Diseases (ICD)-9-CM and ICD-10-CM diagnosis codes. Abdominal paracenteses were identified using corresponding procedure codes. We quantified (1) annual numbers and rates of ED visits with LCAH and abdominal paracentesis relative to all ED visits, (2) distributions of three clinical subgroups of LCAH patients with major clinical complications (CirMCC), clinical comorbidities (CirCC), or without complications/comorbidities (CirWC), and (3) annual proportions of LCAH encounters undergoing versus not undergoing paracentesis.

Results

From 2012 to 2021, there were 214,412 ED encounters with LCAH and 51,683 abdominal paracenteses. The rate of LCAH among all ED visits increased from 0.0030% in 2012 to 0.0287% in 2021, and the rate of abdominal paracentesis among all ED visits rose from 0.0017% to 0.0071% over the same period. Among patients with LCAH, the proportion undergoing paracentesis decreased over time, from 56.9% in 2012 to 24.8% in 2021. CirMCC remained the largest subgroup across the study period, while CirCC and CirWC comprised smaller but relatively stable proportions.

Conclusion

Across a decade of NEDS data, LCAH-related ED visits and absolute numbers of abdominal paracenteses increased, while the proportion of LCAH patients undergoing paracentesis declined. CirMCC, CirCC, and CirWC subgroups maintained stable relative frequencies. These findings suggest evolving patterns in the ED management of liver disease-related ascites and highlight the importance of anticipating procedural demand, staffing needs, and preventive strategies for liver disease.

## Introduction

An abdominal paracentesis, also known as an abdominal tap or ascites tap, is a standard and minimally invasive medical procedure in which a thin plastic catheter or needle is inserted into the peritoneal cavity to remove excess intraperitoneal fluid (ascites) for diagnostic and/or therapeutic purposes [1-2]. Approximately 150,000 abdominal paracenteses are performed annually in the United States [3]. The procedure typically takes about an hour and, in uncomplicated cases, patients can usually resume their regular routines within one to two days [4]. When performed with appropriate technique, abdominal paracentesis is associated with a relatively low risk of major complications and generally does not require substantial long-term lifestyle modification solely as a result of the procedure [5]. Common indications include abdominal pain or discomfort, visible abdominal distension, and dyspnea due to increased intra-abdominal pressure [6].

Ascites is defined as the pathological accumulation of fluid within the peritoneal cavity, most commonly as a consequence of liver cirrhosis or alcoholic hepatitis, although it can also be associated with kidney failure, tuberculosis, and certain malignancies [7]. Symptoms of ascites include progressive abdominal swelling, unintended weight gain, bloating, nausea, vomiting, lower-extremity edema, and subjective sluggishness or fatigue [8]. While clinical examination may be sufficient to suspect ascites, it is usually confirmed with imaging such as abdominal ultrasound or computed tomography (CT) to characterize the extent of fluid accumulation [9-11].

Liver cirrhosis is one of the primary causes of ascites requiring abdominal paracentesis. Cirrhosis leads to portal hypertension and impaired hepatocellular function, both of which contribute to fluid accumulation in the peritoneal cavity [12-13]. Portal hypertension, in combination with decreased hepatic synthetic capacity and reduced albumin production, promotes sodium and water retention and the formation of transudative ascitic fluid [14]. Cirrhosis affects approximately 0.27% of the US population and contributes to roughly 200,000 hospitalizations per year [15]. A large proportion of these hospitalizations involve the need for abdominal paracentesis [16]. The development of ascites in cirrhosis is associated with a two-year survival of about 50% and a five-year survival of 30-40% [17]. Management focuses on treating the underlying etiology and implementing measures such as dietary sodium restriction, diuretics, therapeutic paracentesis, placement of shunts or transjugular intrahepatic portosystemic shunts (TIPS), and, in appropriate candidates, liver transplantation [18]. Because cirrhotic ascites is often recurrent, some patients require repeated paracenteses ranging in frequency from once in a lifetime to several times per week, depending on disease severity and treatment response [19].

Cirrhosis is characterized by the replacement of normal hepatic parenchyma with fibrotic and nodular tissue [20]. This architectural distortion contributes to portal hypertension and progressive hepatic dysfunction, both of which drive the development of ascites [21]. Major etiologies include chronic harmful alcohol use, non-alcoholic fatty liver disease, chronic hepatitis B and C, and autoimmune liver disease [22]. Symptoms may range from nonspecific complaints such as weakness, fatigue, anorexia, and mild abdominal discomfort to more severe manifestations, including abdominal swelling, gastrointestinal bleeding, jaundice, and hepatic encephalopathy in decompensated disease [23-24]. Diagnosis is typically based on a combination of clinical findings, laboratory testing (e.g., complete blood count, liver enzymes, coagulation parameters, and electrolyte panels), imaging (ultrasound or CT), and, when indicated, liver biopsy [23,25]. The natural history of chronic liver disease generally involves progression from hepatitis and early fibrosis to cirrhosis and, ultimately, liver failure if left untreated [26]. Survival varies widely, commonly estimated between two and 16 years depending on disease stage, comorbidities, and adherence to treatment [27]. Although cirrhosis is a chronic condition, interventions such as alcohol cessation, dietary and lifestyle modifications, antiviral therapies, and timely liver transplantation can slow progression and improve outcomes [23,28].

Alcoholic hepatitis is another major contributor to LCAH and a prominent indication for abdominal paracentesis in the context of cirrhotic ascites [29]. It is a severe inflammatory form of alcoholic liver disease caused by prolonged heavy alcohol intake or recurrent binge drinking [29]. Patients may present with right upper quadrant or epigastric pain, tenderness over the liver, unintended weight loss, weakness, persistent fever, nausea, and jaundice [29]. Diagnosis is generally established by clinical features, laboratory abnormalities, and imaging, with liver biopsy reserved for selected cases [29-30]. Management depends on severity and includes complete abstinence from alcohol, nutritional support, pharmacologic therapy when indicated, and treatment of complications; progression from alcoholic hepatitis to cirrhosis may occur if the underlying behavior persists [31]. Although liver cirrhosis and alcoholic hepatitis (LCAH) are the two most common causes of ascites requiring paracentesis, other conditions, such as hepatobiliary carcinoma, can also lead to ascites [32]. Spontaneous bacterial peritonitis, by contrast, is a complication of pre-existing cirrhotic ascites and is an important indication for prompt diagnostic paracentesis rather than a primary cause of fluid accumulation [32].

While abdominal paracenteses are routinely performed procedures, complications and comorbidities may be present in a substantial proportion of patients [33-34]. For the purposes of this study, we categorized LCAH patients into three subgroups. Patients with major clinical complications (CirMCC) include patients presenting to the emergency department (ED) with LCAH who also had major acute clinical complications in the ED, such as sepsis, malignancy, renal failure, or gastrointestinal hemorrhage, based on NEDS comorbidity flags and diagnostic codes. Patients with clinical comorbidities (CirCC) include patients with LCAH and chronic clinical comorbidities such as diabetes, hypertension, or chronic kidney disease, but without major acute complications. Patients without complications/comorbidities (CirWC) refer to patients presenting with LCAH without any identified comorbidities or complications, representing the lowest-acuity subgroup.

Because liver cirrhosis and ascites often present with distressing symptoms such as sudden abdominal pain, progressive swelling, vomiting, or shortness of breath, patients frequently present to the ED for evaluation [35]. Following a history, physical examination, imaging, and laboratory testing, abdominal paracentesis is often required for diagnostic clarification and symptomatic relief [35-36]. Despite the central role of paracentesis in this setting, the temporal patterns and comparative utilization of abdominal paracentesis in EDs have been under-analyzed. Understanding these trends can inform healthcare resource allocation, improve patient care outcomes, and help characterize broader public health patterns in liver disease and procedural intervention.

Accordingly, this study used NEDS data from 2012 to 2021 to describe temporal trends in ED visits for LCAH, quantify the utilization of abdominal paracentesis in this population, and compare patterns across CirMCC, CirCC, and CirWC subgroups.

## Materials and methods

This retrospective observational study utilized data from the National Emergency Department Sample (NEDS) from 2012 to 2021 to analyze ED visits involving abdominal paracentesis in the United States. NEDS is part of the Healthcare Cost and Utilization Project and is the largest all-payer ED database in the US, containing a stratified sample of hospital-based ED encounters from participating states and the District of Columbia. It provides de-identified, encounter-level information on patient demographics, diagnoses, procedures, and hospital characteristics.

We included ED encounters for adults aged 18 years and older with a primary or secondary diagnosis of liver cirrhosis or alcoholic hepatitis (LCAH). LCAH diagnoses were identified using ICD-9-CM codes (e.g., 571.2, 571.5, 571.6) and ICD-10-CM codes (e.g., K70.30, K70.31, K74.60, K74.69), which correspond to alcoholic hepatitis and various forms of cirrhosis. The study included all eligible encounters recorded between 2012 and 2021. Encounters were excluded if they lacked essential demographic information or did not occur in a hospital-based ED.

Abdominal paracentesis procedures were identified using applicable ICD-9-CM and ICD-10-PCS procedure codes specific to paracentesis of the peritoneal cavity. Each ED encounter was classified as either having or not having an abdominal paracentesis performed during that visit, allowing comparison of patients with LCAH who did and did not undergo the procedure.

To examine clinical heterogeneity, LCAH encounters were categorized into three mutually exclusive subgroups using NEDS comorbidity flags and diagnostic codes [33-34]: CirMCC (LCAH encounters in which patients had major acute clinical complications in the ED, such as sepsis, malignancy, renal failure, or gastrointestinal hemorrhage); CirCC (LCAH encounters in which patients had chronic clinical comorbidities, such as diabetes, hypertension, or chronic kidney disease, but no major acute complications); and CirWC (LCAH encounters in which patients had LCAH without identified comorbidities or complications, representing the lowest-acuity subgroup).

We performed three main analyses using NEDS data. First, we calculated annual totals of ED visits with LCAH and annual totals of abdominal paracenteses from 2012 to 2021 and computed descriptive statistics. Second, we calculated annual rates of ED visits with LCAH and annual rates of abdominal paracenteses as proportions of total ED visits in NEDS for each year and compared these rates over time. Third, we calculated annual percentages of LCAH encounters classified as CirMCC, CirCC, and CirWC and determined the proportions of LCAH patients undergoing and not undergoing paracentesis for each year. Because this was a descriptive, exploratory study using administrative data, we focused on summarizing observed patterns rather than performing extensive multivariable modeling. Standard statistical software capable of handling large, weighted administrative datasets was used for all analyses.

## Results

Between 2012 and 2021, a total of 214,412 ED encounters with LCAH were identified. Over the same period, 51,683 abdominal paracenteses were performed in the ED.

When evaluated relative to all ED visits in NEDS, the rate of LCAH increased from 0.0030% in 2012 to 0.0287% in 2021. Intermediate annual values included 0.0117% in 2013, 0.0124% in 2014, a dip back to 0.0030% in 2015, and then a generally rising trend from 0.0127% in 2016 to 0.0210% in 2018, 0.0206% in 2019, and 0.0265% in 2020 before reaching 0.0287% in 2021. The rate of abdominal paracentesis among all ED visits followed a similar broad pattern, starting at 0.0017% in 2012, remaining relatively stable in 2013 (0.0020%) and 2014 (0.0019%), dipping to a low of 0.0009% in 2015, then increasing to 0.0035% in 2016, 0.0044% in 2017, and 0.0057% in 2018. The rate subsequently fluctuated around this higher level, measuring 0.0048% in 2019, 0.0058% in 2020, and reaching a high of 0.0071% in 2021. These annual totals and rates for ED visits with LCAH and for abdominal paracenteses, along with corresponding denominators of total ED visits, are summarized in Table 1.

**Table 1:** Table showcasing total ED visits, the total number of patients presenting to the ED with LCAH, and the total number of abdominal paracenteses performed, along with the rates of the total number of patients presenting to the ED with LCAH and the total number of abdominal paracenteses relative to total ED visits on an annual basis.

Year	Total ED Visits	Total ED patients with LCAH N (%)	Total Number of Abdominal Paracenteses Performed in the ED N (%)
2012	134,399,179	4,069 (0.0030%)	2,315 (0.0017%)
2013	134,869,015	15,738 (0.0117%)	2,636 (0.0020%)
2014	137,807,901	17,025 (0.0124%)	2,608 (0.0019%)
2015	143,469,670	4,277 (0.0030%)	1,333 (0.0009%)
2016	144,842,742	18,403 (0.0127%)	5,101 (0.0035%)
2017	144,814,803	26,135 (0.0180%)	6,440 (0.0044%)
2018	143,454,430	30,056 (0.0210%)	8,175 (0.0057%)
2019	143,432,284	29,492 (0.0206%)	6,878 (0.0048%)
2020	123,278,165	32,730 (0.0265%)	7,121 (0.0058%)
2021	126,968,321	36,487 (0.0287%)	9,076 (0.0071%)

When restricting the analysis to patients presenting to the ED with LCAH, the proportion undergoing abdominal paracentesis displayed substantial variability over time. In 2012, 2,315 of 4,069 LCAH encounters (56.9%) involved paracentesis, whereas 1,754 encounters (43.1%) did not. After 2012, the proportion of LCAH patients undergoing paracentesis generally decreased, with notable year-to-year fluctuations. For example, in 2013, only 16.8% of LCAH encounters involved paracentesis; in 2014, 15.4%; in 2015, 31.2%; and from 2016 onward, annual proportions ranged from approximately 21.8% to 27.8%, ending at 24.8% in 2021. Thus, by the end of the study period, LCAH patients were considerably less likely to undergo paracentesis during their ED visit than at the beginning of the decade, despite increasing absolute numbers of LCAH encounters and procedures.

**Figure 1:**
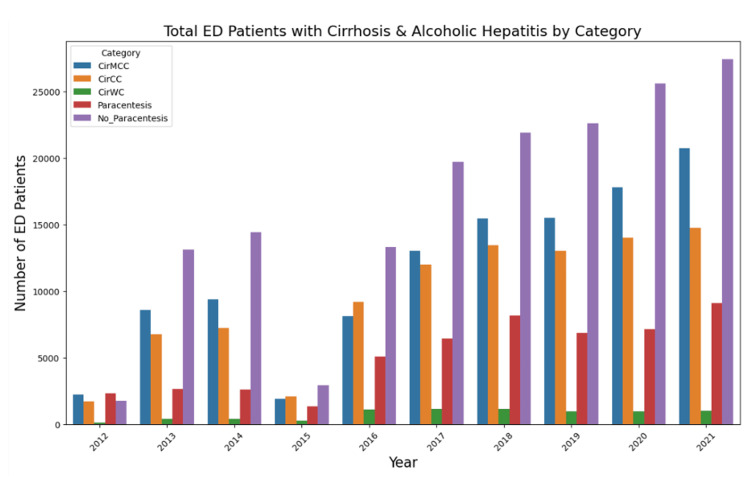
Seaborn plot comparing the total number of patients presenting to the ED with LCAH who underwent an abdominal paracentesis, the total number of patients presenting to the ED with LCAH who did not undergo an abdominal paracentesis, CirMCC patients, CirCC patients, and CirWC patients.

When LCAH encounters were categorized into CirMCC, CirCC, and CirWC, the CirMCC subgroup consistently represented the largest share of LCAH encounters across the study period. For example, CirMCC accounted for 54.9% of LCAH encounters in 2012, dipped to 44.6% in 2015, and rose to 56.8% by 2021. Although there was year-to-year variability, CirMCC proportions remained in a relatively narrow range, with a total spread of about 12.8 percentage points over the decade. The CirCC subgroup followed a roughly unimodal pattern, representing 41.9% of LCAH encounters in 2012, increasing to 49.9% in 2016, then gradually declining to 40.4% by 2021. Overall, CirCC proportions varied within a range of approximately 9.5 percentage points. The CirWC subgroup, representing patients with LCAH and no documented complications or comorbidities, consistently accounted for the smallest proportion of LCAH encounters. Its annual frequency ranged from 2.3% to 6.2%, starting at 3.3% in 2012, peaking at 6.2% in 2015, and declining to 2.7% in 2021. Despite these fluctuations, the relative ordering of the three subgroups remained constant: CirMCC was the most prevalent, CirCC the second most prevalent, and CirWC the least prevalent across all years. Together with the increasing absolute number of LCAH encounters, these trends indicate that the mix of high-, intermediate-, and lower-acuity LCAH presentations remained broadly stable over time, even as overall volumes increased. The annual numbers and percentages of LCAH encounters with and without paracentesis, along with the annual counts and percentages for CirMCC, CirCC, and CirWC, and the corresponding paracentesis and non-paracentesis counts, are presented in Table 2 and visualized in Figure 1.

**Table 2:** Table showcasing total number of ED patients presenting with LCAH, CirMCC patients, CirCC patients, CirWC patients, total number of abdominal paracenteses performed on patients presenting to the ED with LCAH, and patients who presented to the ED with LCAH who did not undergo an abdominal paracentesis. Additionally, rates for CirMCC, CirCC, CirWC, abdominal paracentesis performed, and no abdominal paracentesis performed were calculated relative to total ED patients with LCAH on an annual basis.

Year	Total ED patients with LCAH	CirMCC N (%)	CirCC N (%)	CirWC N (%)	Abdominal Paracentesis Performed N (%)	No Abdominal Paracentesis Performed N (%)
2012	4,069	2,234 (54.9%)	1,702 (41.9%)	133 (3.3%)	2,315 (56.9%)	1,754 (43.1%)
2013	15,738	8,561 (54.3%)	6,774 (43.05%)	403 (2.5%)	2,636 (16.8%)	13,102 (83.2%)
2014	17,025	9,383 (55.2%)	7,252 (42.5%)	390 (2.3%)	2,608 (15.4%)	14,417 (84.6%)
2015	4,277	1,911 (44.6%)	2,105 (49.2%)	261 (6.2%)	1,333 (31.2%)	2,944 (68.8%)
2016	18,403	8,099 (44.01%)	9,189 (49.9%)	1,115 (6.1%)	5,101 (27.8%)	13,302 (72.2%)
2017	26,135	13,007 (49.7%)	11,977 (46%)	1,151 (4.5%)	6,440 (24.7%)	19,695 (75.3%)
2018	30,056	15,445 (51.3%)	13,464 (44.7%)	1,147 (3.8%)	8,175 (27.2%)	21,881 (72.8%)
2019	29,492	15,496 (52.5%)	13,013 (44.12%)	983 (3.4%)	6,878 (23.3%)	22,614 (76.6%)
2020	32,730	17,768 (54.3%)	14,005 (42.8%)	957 (2.9%)	7,121 (21.8%)	25,609 (78.2%)
2021	36,487	20,716 (56.8%)	14,759 (40.4%)	1,012 (2.7%)	9,076 (24.8%)	27,411 (75.2%)

## Discussion

This study provides a descriptive, national-level overview of temporal trends in ED utilization of abdominal paracentesis among patients with LCAH using NEDS data from 2012 to 2021. Over this decade, both ED encounters for LCAH and the absolute number of abdominal paracenteses increased. When viewed relative to total ED visits, the rates of LCAH and paracentesis also rose, with a transient dip in 2015 followed by a steady upward trajectory. These findings align with broader reports of increasing chronic liver disease and alcohol-related harm in the United States over recent years [37-40].

At the same time, among patients presenting with LCAH, the proportion undergoing paracentesis decreased. This pattern suggests evolving use of paracentesis within a growing pool of LCAH patients. Multiple factors may contribute. Rising prevalence of chronic liver disease, driven in part by increasing alcohol consumption, binge drinking, and metabolic risk factors, likely increases the number of LCAH encounters [38-42]. However, changes in treatment paradigms, improved outpatient follow-up, and expanded use of alternative therapies (e.g., optimized diuretic regimens, TIPS placement, or scheduled outpatient paracenteses) may have reduced the proportion of LCAH patients who undergo paracentesis during a given ED visit [39-43].

Although the increasing number of LCAH encounters and abdominal paracenteses has important implications for ED capacity and resource planning, the decline in the proportion of LCAH patients undergoing paracentesis may represent more selective procedure use. It is possible that clinicians are reserving paracentesis for patients with clear diagnostic or therapeutic indications (e.g., suspected spontaneous bacterial peritonitis or severe symptomatic ascites), while managing others medically and arranging timely outpatient care [41-43]. At the same time, underuse of paracentesis in patients with guideline-based indications, particularly for suspected spontaneous bacterial peritonitis, could have adverse consequences. Future work linking administrative data with clinical variables would help clarify whether the observed decline reflects appropriate selectivity or potential underutilization in high-risk subgroups.

The relatively stable distributions of CirMCC, CirCC, and CirWC across the study period add important context to these trends. CirMCC, representing LCAH encounters with major acute complications, remained the largest subgroup throughout, underscoring the high acuity of many patients presenting with advanced liver disease [44-45]. Conditions such as malignancy, severe jaundice, gastrointestinal bleeding, and sepsis carry serious prognostic implications and often necessitate intensive inpatient care [17,23,44]. The persistence of a large CirMCC subgroup, even as total LCAH encounters rise, suggests that EDs must continue to be prepared for frequent high-complexity presentations requiring rapid stabilization, multidisciplinary coordination, and specialized hepatology input.

CirCC represents patients with LCAH and chronic comorbidities (e.g., diabetes, hypertension, chronic kidney disease) but without major acute complications. Its relative stability over time is consistent with broader trends in the increasing prevalence of chronic cardiometabolic conditions [46-47]. These comorbidities complicate management, increase the risk of decompensation, and may require simultaneous attention alongside liver disease. Public health efforts targeting lifestyle modification, early detection of metabolic disease, and primary prevention remain important for reducing the overlapping burdens of chronic liver disease and its comorbidities [48-49].

CirWC, the smallest subgroup, represents LCAH encounters without documented complications or comorbidities. Ideally, one might hope to see this subgroup increase over time as a reflection of earlier detection and more widespread preventive care. Instead, its relatively low and stable proportion suggests that a large share of patients with LCAH still present with significant comorbidity or acute complications. This highlights opportunities for earlier outpatient identification of liver disease and more proactive management to prevent progression to advanced, decompensated stages.

Overall, the combination of increasing LCAH encounters, rising absolute paracentesis counts, decreasing paracentesis proportions among LCAH patients, and stable CirMCC/CirCC/CirWC distributions points to an ED environment in which liver disease-related presentations are becoming more common but are managed with a slightly different procedural profile. From a systems perspective, these patterns underscore the importance of ensuring sufficient ED staffing and procedural expertise for abdominal paracentesis, maintaining ready access to ultrasound guidance and sterile equipment, and strengthening pathways to hepatology and interventional radiology services [43-45]. At the same time, they highlight the need for robust public health and primary care efforts targeting alcohol use, metabolic disease, and other modifiable liver disease risk factors [37-42,46-50].

Finally, ED overcrowding and prolonged wait times continue to be major concerns nationally, and rising procedural demand may exacerbate these challenges if not anticipated and planned for appropriately [51]. Allocating adequate personnel and equipment to support paracentesis and related procedures, and integrating them into streamlined care pathways, may help mitigate bottlenecks and maintain timely access to care [52]. Educational initiatives aimed at both clinicians and the public regarding liver disease, appropriate indications for paracentesis, and preventive strategies have been shown to improve knowledge and may reduce downstream disease burden [53-54].

Limitations

Several limitations should be considered when interpreting these findings. First, this retrospective study relies on administrative data from NEDS, which are subject to potential coding errors, misclassification, and incomplete documentation [55-56]. Second, NEDS is an encounter-level database that does not allow linkage of multiple visits by the same patient; therefore, repeat ED visits or procedures for a single individual are counted as separate encounters. Third, the dataset does not capture all potentially relevant clinical variables, such as laboratory values, imaging findings, hemodynamic status, or detailed indications for paracentesis, limiting assessment of disease severity and the appropriateness of procedure use. Fourth, generalizability may be constrained by the fact that NEDS includes hospital-based EDs and may not capture all healthcare settings or patient populations. Finally, the marked dip observed in 2015 likely reflects the transition from ICD-9-CM to ICD-10-CM diagnosis coding, which may have led to underreporting or inconsistent coding of LCAH and paracentesis during that year [55-56].

## Conclusions

This study, using NEDS data from 2012 to 2021, provides a national-level description of temporal trends in ED utilization of abdominal paracentesis among patients with LCAH in the United States. The rate of LCAH presentations and the absolute number of abdominal paracenteses performed in EDs increased over the decade. Although the rate of paracentesis relative to total ED visits rose, the proportion of LCAH patients undergoing paracentesis declined, suggesting evolving management strategies for liver disease and ascites in the ED. Throughout the study period, CirMCC, CirCC, and CirWC subgroups maintained relatively stable relative frequencies, with CirMCC consistently representing the largest subgroup.

These findings deepen our understanding of abdominal paracentesis utilization and highlight opportunities to improve ED resource allocation, procedural capacity, and linkage to specialty care. They also underscore the importance of preventive strategies, early outpatient management, and public health interventions aimed at reducing the burden of chronic liver disease and its complications. Further research is warranted to explore patient-level determinants of paracentesis use and to assess how changes in practice patterns affect outcomes in this growing population.
